# Development of organ‐specific autoimmunity by dysregulated Aire expression

**DOI:** 10.1111/imcb.12546

**Published:** 2022-04-09

**Authors:** Hitoshi Nishijima, Mizuki Sugita, Natsuka Umezawa, Naoki Kimura, Hirokazu Sasaki, Hiroshi Kawano, Yasuhiko Nishioka, Minoru Matsumoto, Takeshi Oya, Koichi Tsuneyama, Junko Morimoto, Mitsuru Matsumoto

**Affiliations:** ^1^ Division of Molecular Immunology Institute for Enzyme Research Tokushima University Tokushima Japan; ^2^ Department of Rheumatology Graduate School of Medical and Dental Sciences Tokyo Medical and Dental University Tokyo Japan; ^3^ Department of Respiratory Medicine and Rheumatology Tokushima University Graduate School of Biomedical Sciences Tokushima Japan; ^4^ Department of Molecular Pathology Tokushima University Graduate School of Biomedical Sciences Tokushima Japan; ^5^ Department of Pathology and Laboratory Medicine Tokushima University Graduate School of Biomedical Sciences Tokushima Japan; ^6^ Present address: Department of Immunology Tokyo Medical University Tokyo Japan

**Keywords:** Aire, autoimmune disease, EAE, mTEC, polymyositis, self‐tolerance

## Abstract

Deficiency for AIRE/Aire in both humans and mice results in the development of organ‐specific autoimmune disease. We tested whether augmented and/or dysregulated AIRE/Aire expression might be also prone to the breakdown of self‐tolerance. To define the effect of augmented Aire expression on the development of autoimmunity, antigen‐specific clonal deletion and production of clonotypic regulatory T cells (Tregs) in the thymus were examined using mice expressing two additional copies of Aire in a heterozygous state (3xAire‐knockin mice: 3xAire‐KI). We found that both clonal deletion of autoreactive T cells and production of clonotypic Tregs in the thymus from 3xAire‐KI were impaired in a T‐cell receptor‐transgenic system. Furthermore, 3xAire‐KI females showed higher scores of experimental autoimmune encephalomyelitis induced by myelin oligodendrocyte glycoprotein than wild‐type littermates, suggesting that augmented Aire expression exacerbates organ‐specific autoimmunity under disease‐prone conditions. In humans, we found that one patient with amyopathic dermatomyositis showed CD3^–^CD19^–^ cells expressing AIRE in the peripheral blood before the treatment but not during the remission phase treated with immunosuppressive drugs. Thus, not only loss of function of AIRE/Aire but also augmented and/or dysregulated expression of AIRE/Aire should be considered for the pathogenesis of organ‐specific autoimmunity. We suggest that further analyses should be pursued to establish a novel link between organ‐specific autoimmune disease and dysregulated AIRE expression in clinical settings.

## Graphical abstract

Mice expressing augmented Aire from authentic Aire‐expressing cells showed higher experimental autoimmune encephalomyelitis scores than wild‐type littermates, suggesting that augmented Aire expression exacerbates organ‐specific autoimmunity. This was associated with the defect in the antigen‐specific clonal deletion and production of clonotypic regulatory T cells in the thymus. Interestingly, one patient with amyopathic dermatomyositis showed CD3^–^CD19^–^ cells expressing AIRE in the peripheral blood before the treatment but not during the remission phase, suggesting a novel link between organ‐specific autoimmunity and dysregulated AIRE expression also in clinical settings.

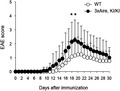

## INTRODUCTION

Organ‐specific autoimmune diseases are caused by the misguided immune responses against self‐antigens expressed from particular cell types in the body. Although target antigens recognized by autoantibodies and/or autoreactive T cells have been identified in some cases, a fundamental question of how autoimmune responses arise in the first place remains largely unknown. In this regard, elucidation of the molecular pathogenesis of autoimmune polyendocrinopathy‐candidiasis‐ectodermal dystrophy (OMIM 240300) would help to solve the enigma because it is a monogenic disease with mutations of the *AIRE*, enabling us to use an animal model for the study.^1^, ^2^, ^3^, ^4^ Aire is predominantly expressed from thymic epithelial cells in the medulla (mTECs), and transcriptomic analyses using Aire‐deficient mice demonstrated that Aire controls wide varieties of genes in mTECs. Among these Aire‐dependent genes, much attention has been paid to the genes of tissue‐restricted antigens (TRAs) because reduced expression of TRAs in Aire‐deficient mTECs would result in the impaired elimination of autoreactive T cells and/or the impaired production of regulatory T cells (Tregs) in an antigen‐specific manner.^5^, ^6^, ^7^, ^8^ Besides mTECs, Aire is expressed from antigen‐presenting cells (APCs) in the periphery, and their complementing role to the thymic tolerance mechanisms has been demonstrated.^9^ According to these scenarios, augmented Aire expression would mitigate the organ‐specific autoimmunity. However, this may not be necessarily the case for both animal models and human patients.

## RESULTS

### The paradoxical propensity for the development of autoimmunity by augmented Aire expression

**Figure 1:**
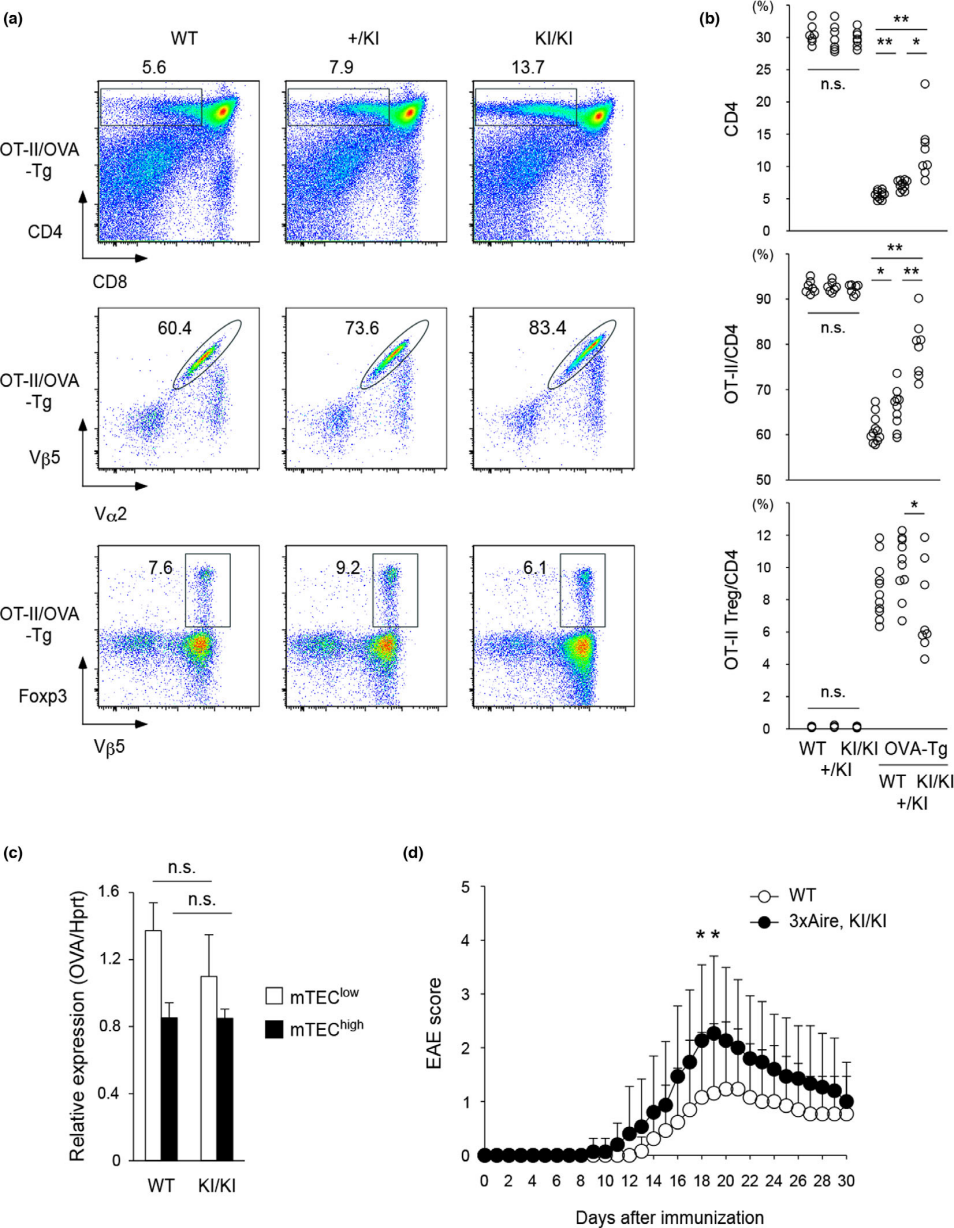
A propensity for organ‐specific autoimmunity by augmented Aire expression. **(a)** Impaired clonal deletion of CD4 ^+^ OT‐II T cells in the thymus upon crossing with RIP‐OVA Tg on the heterozygous and homozygous 3xAire‐KI backgrounds. After gating for CD4 single‐positive T cells (top), clonotypic T cells were assessed using anti‐Vα2 and anti‐Vβ5 mAbs (middle). Impaired production of antigen‐specific Tregs in the thymus by additive expression of Aire in OT‐II Tg crossed with RIP‐OVA Tg (bottom). **(b)** Summary of the results shown in **a**. Mice at 3–4 weeks of age for each genotype (WT, *n* = 7; +/KI, *n* = 7; KI/KI, *n* = 7 for OT‐II Tg and WT, *n* = 11; +/KI, *n* = 10; KI/KI, *n* = 8 for OT‐II/OVA‐Tg) were analyzed in a total of nine repeated experiments. *P < 0.05; **P < 0.01. **(c)** Levels of OVA expression from isolated mTEC^low^ (white) and mTEC^high^ (black) populations in RIP‐OVA Tg on WT (*n* = 3) or homozygous 3xAire‐KI background (*n* = 3) were examined by real‐time PCR in a total of three repeated experiments. The expression level of *Hprt* was used as an internal control for RNAs. Data are shown as the averages and standard error of the means obtained from three repeated experiments by preparing triplicate samples for each experiment. **(d)** EAE scores of female mice from WT (*n* = 13) and homozygous 3xAire‐KI (*n* = 15) were obtained from a total of two repeated experiments. *P < 0.05. EAE, experimental autoimmune encephalomyelitis; KI, knock in; mAb, monoclonal antibody; mTEC, thymic epithelial cells in the medulla; OVA, ovalbumin; RIP, rat insulin promoter; n.s., not significant; Tg, transgenic; Tregs, regulatory T cells; WT, wild type.

We wanted to test whether augmented Aire expression alters the processes for the negative selection and Treg production in the thymus using 3xAire‐KI in which two additional copies of mouse Aire complementary DNA were introduced into the *Aire* locus. 3xAire‐KI showed augmented Aire expression from authentic Aire‐expressing mTECs detected by flow cytometry and immunohistochemical analysis.^10^ We examined the thymic tolerance event by employing a T‐cell receptor (TCR)‐transgenic (Tg) model.^11^ Ovalbumin (OVA)‐specific TCR‐Tg (OT‐II Tg) were crossed with rat insulin promoter (RIP)‐driven OVA Tg (RIP‐OVA Tg) on heterozygous and homozygous 3xAire‐KI backgrounds. With this experimental system, Aire‐deficient mice showed impaired clonal deletion and reduced production of clonotypic Tregs.^11^ Unexpectedly, 3xAire‐KI also showed impaired deletions of clonotypic CD4^+^ T cells in a dose‐dependent manner of Aire: the more Aire, the less clonal deletion (Figure 1a, b, top and middle). In this TCR‐Tg model, Vβ5^+^ clonotypic Foxp3^+^ Tregs were generated in the thymus upon crossing with RIP‐OVA Tg.^11^ Fewer percentages of clonotypic Tregs were generated on a homozygous 3xAire‐KI background than on a heterozygous background (Figure 1a, b, bottom), although absolute numbers of clonotypic Tregs were not decreased (data not shown) because total CD4^+^ T‐cell numbers were increased as a result of the impaired clonal deletion in homozygous 3xAire‐KI (Figure 1a, b, top). Thus, both clonal deletion of autoreactive T cells and production of clonotypic Tregs in the thymus were affected by the augmented Aire expression. Of note, expression levels of OVA from both immature (CD80^low^MHC‐II^low^: mTEC^low^) and mature mTECs (CD80^high^MHC‐II^high^: mTEC^high^) remained unchanged in RIP‐OVA Tg on the homozygous 3xAire‐KI background (Figure 1c), suggesting that impaired thymic tolerance with this TCR model was independent of the gene dosage effect of model self‐antigen of OVA.

3xAire‐KI had indistinguishable numbers of total thymocytes with normal ratios of CD4 to CD8, and percentages of thymic Tregs among CD4^+^ T cells were comparable with those from wild type in a polyclonal (non‐TCR‐Tg) setting.^10^ Both heterozygous and homozygous 3xAire‐KI showed no spontaneous autoimmune phenotypes upon pathological examination (Hitoshi Nishijima, Minoru Matsumoto and Mitsuru Matsumoto, unpublished observation). Of note, autoimmune phenotypes of Aire‐deficient mice are rather mild on a C57BL/6 background.^12^, ^13^ However, Aire‐deficient mice showed higher susceptibility to the antigen‐induced autoimmune model of experimental autoimmune encephalomyelitis (EAE) than the wild‐type mice in an age‐dependent fashion.^14^ Given that augmented Aire expression resulted in impaired thymic tolerance using a TCR‐Tg model as described above, we asked whether 3xAire‐KI might be prone to autoimmunity using the EAE model. We immunized mice with MOG and evaluated the clinical scores for the paralysis. Homozygous 3xAire‐KI females but not males (data not shown) at 8–10 weeks of age showed higher EAE scores than the wild type (Figure 1d), suggesting that augmented Aire expression exacerbates organ‐specific autoimmunity under disease‐prone conditions.

### Ectopic AIRE expression from the peripheral blood in a patient with amyopathic dermatomyositis

**Figure 2:**
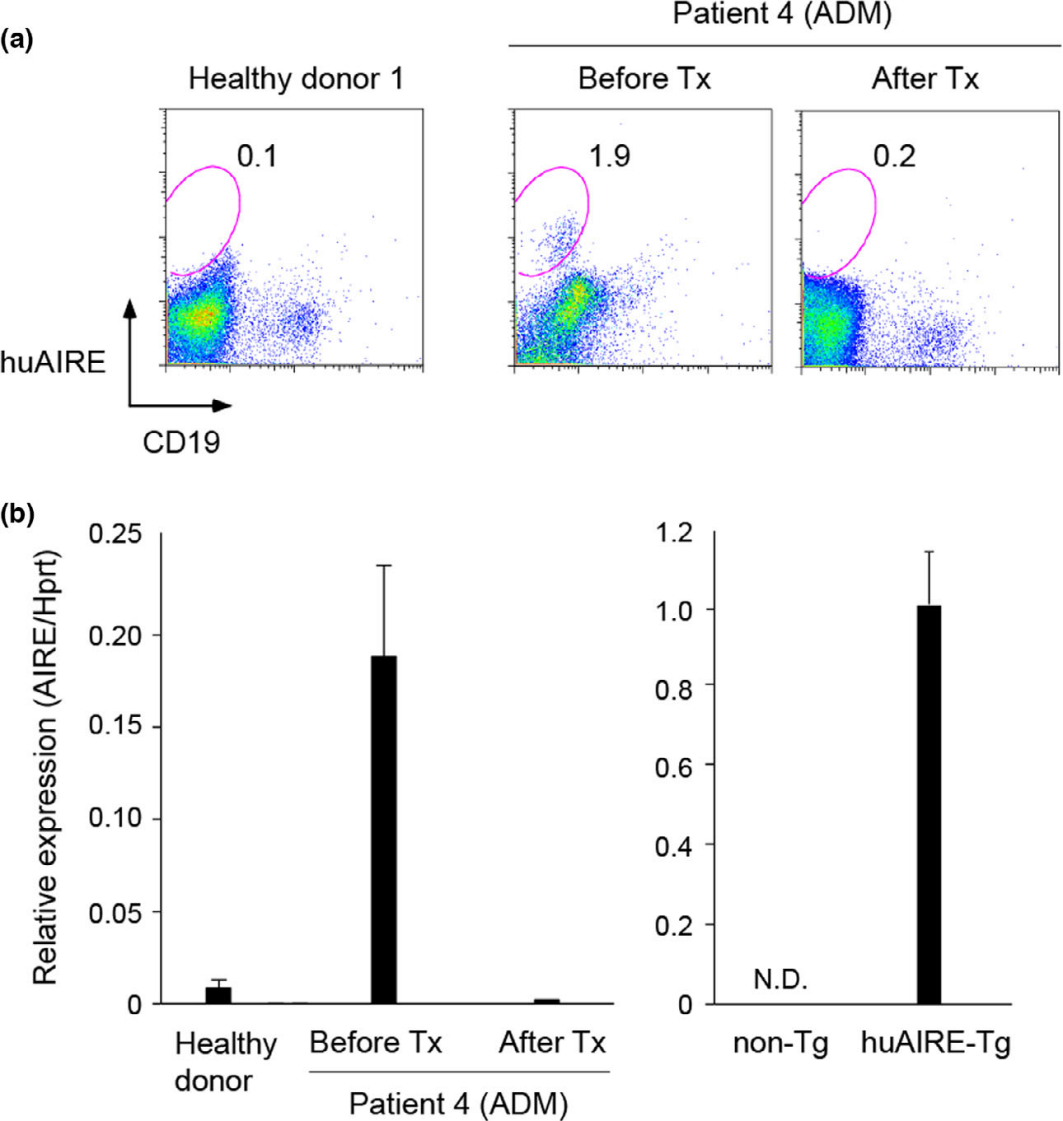
Ectopic AIRE expression in the peripheral blood from patients with organ‐specific autoimmunity. **(a)** Flow cytometric analysis of AIRE expression from peripheral blood. Peripheral blood from a healthy donor served as negative control (left). Peripheral blood from one patient with ADM showed AIRE^+^ cells from CD19^–^ fraction before (Before Tx: center) but not after the treatment (After Tx: right). **(b)** Detection of human AIRE from the peripheral blood mononuclear cells by real‐time PCR. Expression levels of human AIRE were compared before (Before Tx) and after the treatment (After Tx) in Patient 4. Healthy donors served as control (left). The expression level of *Hprt* was used as an internal control for RNAs. Data are shown as the averages and standard error of the means obtained from triplicate samples. One representative result from a total of two experiments is shown. Expression of human AIRE from splenocytes isolated from huAIRE‐Tg was shown as a reference (right). One representative result from a total of more than three experiments is shown. ADM, amyopathic dermatomyositis; ND, not detected; Tg, transgenic.

Previously, we have demonstrated that Tg mice additively expressing human AIRE (huAIRE‐Tg) under the control of MHC‐II promoter paradoxically developed muscle‐specific autoimmunity resembling polymyositis in humans on the autoimmune‐prone background of NOD (non‐obese diabetic).^15^ Knowing that both augmented mouse Aire expression from authentic Aire‐expressing cells (3xAire‐KI) and additive expression of human AIRE in MHC‐II^+^ APCs (i.e. mTECs and bone marrow‐derived APCs such as dendritic cells; huAIRE‐Tg) paradoxically showed a propensity to the development of organ‐specific autoimmunity, we searched for augmented and/or ectopic AIRE expression in human patients with autoimmune disease. Because huAIRE‐Tg exhibited polymyositis‐like autoimmunity, we examined the peripheral blood from the patients with polymyositis and its related diseases as an initial trial with small numbers of the patients. Peripheral blood mononuclear cells were simultaneously stained with anti‐CD19 and anti‐human AIRE monoclonal antibodies (mAbs): the specificity of anti‐human AIRE mAb was confirmed using the splenocytes from huAIRE‐Tg^15^ (Supplementary figure 1a): in addition to many CD19^+^AIRE^+^ cells, small percentages of CD19^–^ cells expressed human AIRE in huAIRE‐Tg (i.e. 3.7% in a squared area of Supplementary figure 1a, right). We found that one patient with amyopathic dermatomyositis (Patient 4) showed CD19^–^ cells expressing AIRE before the treatment (i.e. 1.9% in a circled area of Supplementary figure 1b), whereas two healthy donors and the other five patients (Supplementary table 1) had no AIRE^+^ cells in the peripheral blood. AIRE^+^ cells from Patient 4 were negative for CD3 (data not shown). Interestingly, when the blood from this patient with amyopathic dermatomyositis was re‐examined during the remission phase after the treatment with immunosuppressive drugs, AIRE^+^ cells were not detected anymore (Figure 2a, right). Consistent with flow cytometric analysis, AIRE expression detected by real‐time PCR in the peripheral blood mononuclear cells disappeared after the treatment (Figure 2b, left). We could not further characterize the AIRE^+^ cells found in the initial sample because of the limited material.

## DISCUSSION

Our results with mouse models and one human patient suggested that not only loss of function of AIRE/Aire, but also augmented and/or dysregulated expression of AIRE/Aire should be considered for the pathogenesis of organ‐specific autoimmunity. In this regard, the connection between the increased frequency of autoimmune diseases in Down syndrome and the gene dosage effect of AIRE may merit attention. Down syndrome is caused by the trisomy of chromosome 21 in which *AIRE* is located, and one study reported that AIRE messenger RNA levels were elevated in thymic tissue from patients with Down syndrome,^16^ although there are conflicting reports.^17^, ^18^ Likewise, it would be interesting to examine whether AIRE is overexpressed in some patients with thymomas that are associated with organ‐specific autoimmunity, although AIRE expression has been reported to be absent in most thymoma samples except for the B1 subtype.^19^

How do both absence and dysregulated AIRE/Aire expression render the thymus prone to autoimmune microenvironment? Our single‐cell analyses of TECs from Aire‐augmented (3xAire‐KI) and Aire‐deficient mice revealed altered heterogeneity of mTECs in both cases.^10^ Because the acquisition of the properties of TRA expression in mTECs depends on the maturation status of mTECs,^20^ perturbation of the maturation process either by the loss of AIRE/Aire or by the dysregulated AIRE/Aire expression might account for the impaired expression of TRAs.^10^ Thus, the expression of high levels of TRAs is ensured by the controlled mTEC differentiation program with the appropriate level of AIRE/Aire expression.

RIP‐OVA Tg crossed with OT‐II Tg showed impaired negative selection and Treg production in both Aire‐deficient^11^ and homozygous 3xAire‐KI backgrounds (Figure 1a, b). Given the importance of expression of TRAs for the thymic tolerance mechanisms, it was somewhat unexpected that OVA expression in mTECs was unaltered in both cases^11^ (Figure 1c) when crossed with RIP‐OVA Tg. The results suggested that the expression of TRAs is not the sole determinant of thymic tolerance and other factors may govern the process in this model. We speculate that altered heterogeneity of mTECs caused by the lack of Aire and augmented Aire may change in the expression of tolerance‐relevant genes from mTECs, besides TRAs, such as chemokines^10^ and co‐stimulatory molecules.^21^ Further studies are required to reveal the exact mechanisms underlying Aire‐dependent thymic tolerance. How the defect in the thymic tolerance is linked to the higher susceptibility to EAE in 3xAire‐KI also needs to be clarified.

Although our human studies are preliminary with limited numbers of samples, we have observed one patient with amyopathic dermatomyositis who exhibited the ectopic AIRE expression from peripheral blood that was associated with the disease activity. Although the detailed phenotypic analysis of AIRE^+^ cells in this patient was not performed, we speculate that these cells might be APCs such as dendritic cells^22^, ^23^ because they showed no expression of T‐/B‐cell markers. Recent studies in mice have demonstrated Aire^+^ dendritic cells as an important source of self‐antigens,^23^ and expansion of self‐APCs in this patient might have been paradoxically contributing to the production of autoantibodies including anti‐aminoacyl tRNA synthetase autoantibody together with the activation of autoreactive T cells. Similarly, although we have suggested a link between the ectopic AIRE expression in the peripheral blood and disease activity in this patient, it is possible that other factors such as the treatment of the disease and autoregulation by the immune homeostasis might have contributed to this connection.

Because it was impossible to assess the AIRE expression in the patient’s thymus (Patient 4), we do not know whether ectopic AIRE expression in the peripheral blood reflects the augmented AIRE expression in the thymus or it occurred independently from thymic AIRE expression. In this regard, it might be important to note that huAIRE‐Tg developed polymyositis‐like autoimmunity only when both thymic stromal cells (i.e. mTECs) and peripheral APCs (i.e. dendritic cells) expressed a high amount of human AIRE when assessed using the bone marrow‐chimera experiments.^15^, ^24^ Thus, not only augmented AIRE/Aire expression in the thymus but also dysregulated extrathymic AIRE/Aire expression^9^ may contribute to the development of organ‐specific autoimmunity.

In conclusion, we suggest that further analyses should be pursued to establish a novel link between organ‐specific autoimmune disease and dysregulated AIRE expression in clinical settings. Although we have experienced only a single case so far, more investigation should be performed because AIRE expression can be monitored with conventional flow cytometric analysis using the peripheral blood as demonstrated in this study.

## METHODS

### Mice

Mice with augmented Aire expression from authentic Aire‐expressing cells were generated by homologous recombination in embryonic stem (ES) cells established from C57BL/6 (3xAire‐knockin mice: 3xAire‐KI).^10^ 3xAire‐KI were maintained on a C57BL/6 background. TCR‐Tg mice specific for OVA in the context of I‐A^b^ mice (OT‐II Tg) were purchased from The Jackson Laboratory (Bar Harbor, ME, USA). Tg mice expressing OVA under control of the RIP (RIP‐OVA Tg) were kindly provided by Dr Michael J Bevan (University of Washington, Seattle, WA, USA).^11^, ^15^ The mice were maintained under pathogen‐free conditions and handled in accordance with the Guidelines for Animal Experimentation of Tokushima University School of Medicine.

### Experimental autoimmune encephalomyelitis model

EAE was induced in 8–10‐week‐old females of 3xAire‐KI and wild‐type littermates via subcutaneous immunization with 200 μg of myelin oligodendrocyte glycoprotein (MOG)_35–55_ peptide (MEVGWYRSPFSRVVHLYRNGK) in an emulsion with complete Freund’s adjuvant supplemented with 500 μg of *Mycobacterium tuberculosis* H37RA (catalog number 231141; Merck, Darmstadt, Germany). Mice were injected intraperitoneally with 400 ng of pertussis toxin from *Bordetella pertussis* (catalog number 516560; Merck) in phosphate‐buffered saline on the day of immunization and 2 days after immunization. The mice were examined daily for clinical signs of EAE and scored on a 5‐point scale: 0, no clinical disease; 1, limp tail; 2, hindlimb weakness; 3, complete hindlimb paralysis; 4, hindlimb paralysis and some forelimb paralysis and 5, moribund or dead.

### Thymic epithelial cells and thymocytes

Preparation of TECs and flow cytometric analysis of thymocytes with an FACSAria II equipment (BD Life Sciences, San Jose, CA, USA) were performed as described previously.^10^, ^15^ The mAbs used were anti‐CD45, anti‐Ly51, anti‐CD80, anti‐CD8a, anti‐Foxp3 and anti‐I‐A/I‐E, all purchased from eBioscience (Waltham, MA, USA). Anti‐CD4 and anti‐EpCAM mAbs were from BioLegend (San Diego, CA, USA). Anti‐TCR/Vα2 and anti‐TCR/Vβ5 mAbs were from BD Biosciences (Franklin Lane, NJ, USA). *Ulex europaeus* agglutinin 1 (UEA‐1) was from Vector Laboratories (Burlingame, CA, USA).

### Flow cytometry of human peripheral blood

Mononuclear cells isolated from heparinized peripheral blood were obtained from the patients and healthy donors. Anti‐human CD19 and anti‐human AIRE (clone TM‐724) mAbs were from BD Biosciences and eBioscience, respectively. Anti‐human AIRE mAb was labeled with Alexa Fluor 647 (Thermo Fisher Scientific, Waltham, MA, USA) in our laboratory.

### Real‐time PCR

Real‐time PCR for OVA from CD80^low^MHC‐II^low^ immature and CD80^high^MHC‐II^high^ mature mTECs was performed as described previously.^10^, ^15^ RNAs from the peripheral blood mononuclear cells and TECs were extracted using RNeasy Mini Kits (QIAGEN, Venlo, Netherlands) and converted to complementary DNA with SuperScript III or VILO RT Kits (Invitrogen, Waltham, MA, USA) in accordance with the manufacturer’s instructions. Real‐time PCR for quantification of the OVA, AIRE and Hprt genes was performed as described previously.^15^ The primers and dual‐labeled probes (FAM/TAMRA) used are described in Supplementary table 2.

### Case description

A 61‐year‐old male consulted the hospital with the chief complaint of dyspnea on exertion. The chest X‐ray suggested interstitial pneumonitis. Because he showed Gottron’s papule on his elbows and mechanics’ hands, blood examination was conducted using the anti‐aminoacyl tRNA synthetase autoantibody. He did not suffer from muscle weakness nor elevation of muscle enzymes was observed. He was diagnosed with amyopathic dermatomyositis according to the EULAR/ACR classification criteria.^25^ Prednisolone (70 mg/day) and cyclosporine were suggested as treatments, and his symptoms had gradually disappeared. Prednisolone (4 mg/day) and cyclosporine are being given as maintenance therapy without any recurrence of the symptoms. Peripheral blood was taken before (August 2014) and after the treatment (February 2016), and was kept frozen at –80°C until use for the examination of AIRE‐expressing cells.

## AUTHOR CONTRIBUTION

**Hitoshi Nishijima:** Conceptualization; Data curation; Formal analysis; Investigation; Methodology; Project administration; Writing – original draft; Writing – review & editing. **Mizuki Sugita:** Formal analysis; Investigation. **Natsuka Umezawa:** Formal analysis; Investigation; Project administration; Writing – original draft. **Naoki Kimura:** Formal analysis; Investigation; Project administration. **Hirokazu Sasaki:** Formal analysis; Investigation; Project administration. **Hiroshi Kawano:** Project administration. **Yasuhiko Nishioka:** Project administration; Supervision. **Minoru Matsumoto:** Formal analysis; Investigation. **Takeshi Oya:** Formal analysis; Investigation; Supervision. **Koichi Tsuneyama:** Formal analysis; Investigation; Supervision. **Junko Morimoto:** Formal analysis; Investigation; Methodology. **Mitsuru Matsumoto:** Conceptualization; Formal analysis; Funding acquisition; Investigation; Methodology; Project administration; Supervision; Writing – original draft; Writing – review & editing.

## CONFLICT OF INTEREST

The authors have no conflict of interest to declare.

## Supporting information

Supplementary Material
