# Supplementary material for: Development of organ‐specific autoimmunity by dysregulated Aire expression
Source: Immunol Cell Biol. 2022 Apr 9;100(5):371–7. doi: 10.1111/imcb.12546 (PMC9541787; doi:10.1111/imcb.12546)
Supplement: Supplementary file 1 — Supplementary Material [file IMCB-100-371-s001.pdf]

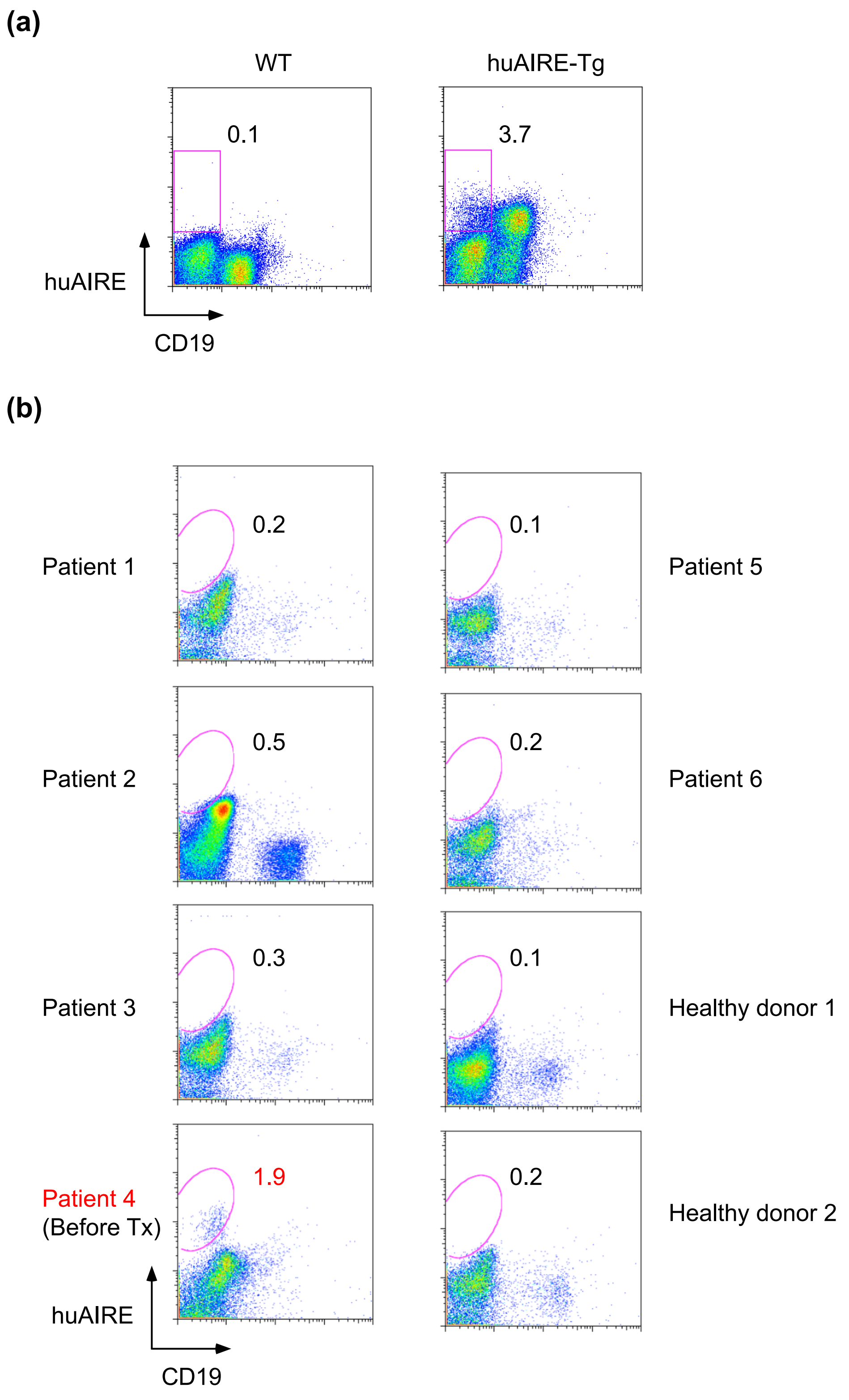

Supplementary figure 1. Detection of AIRE with flow-cytometric analysis.
(a) Detection of human AIRE from splenocytes in huAIRE-Tg with flowcytometric analysis. Wild-type non-Tg mice (WT) served as a negative control. Percentages of $AIRE^{+}CD19^{-}$ cells surrounded by purple squares are indicated. One representative result from a total of more than three experiments is shown.
(b) Flow-cytometric analysis of AIRE expression from peripheral blood. Peripheral blood mononuclear cells from Patient 4 (before the treatment: Before Tx) but not from the other five patients showed $AIRE^{+}$ cells. Peripheral blood from two healthy donors served as control.

Supplementary table 1: List of the patients

Patient	Age	Sex	Diagnosis	Interstitial pneumonia	Autoantibody
1	85	F	Dermatomyositis	—	
2	60	F	Dermatomyositis	—	
3	64	F	Myositis	—	Anti-SRP*
4	61	M	ADM	Severe	Anti-ARS**
5	60	F	Myositis	—	
6	50	F	Dermatomyositis	Mild	

* anti-signal recognition particle antibody

** anti-aminoacyl tRNA synthetase antibody

Supplementary table 2: Primers and probes

Gene	Primer	
OVA	Forward:	5’-CTGTCTGGCATCTCCTCAGC-3’
	Reverse:	5’-TGACCCTACCACCTCTCTGC-3’
AIRE	Forward:	5’-CGGTGGAGGTGAGGCTAGG-3’
	Reverse:	5’-CAGCCGTCACAGCAGATGAG-3’
Mouse Hprt	Forward:	5’-TTCCCTGGTTAAGCAGTACAGC-3’
	Reverse:	5’-CCAACAAAGTCTGGCCTGTATC-3’
Human Hprt	Forward:	5’-GGTCAGGCAGTATAATCCAAAGATG-3’
	Reverse:	5’-AACAAAGTCTGGCTTATATCCAACAC-3’

Gene	Dual-Labeled Probe (FAM/TAMRA)
OVA	5’-TCTCAAGCTGTCCATGCAGCACATGCAG-3’
AIRE	5’-ACGGCACACTCGTCCTCATTCTTCTGGT-3’
Mouse Hprt	5’-TTCGAGAGGTCCTTTTCACCAGCAAGCT-3’
Human Hprt	5’-TCGTGGGGTCCTTTTCACCAGCAAGC-3’
